# Primary Decompressive Craniectomy After Traumatic Brain Injury: A Literature Review

**DOI:** 10.7759/cureus.29894

**Published:** 2022-10-04

**Authors:** Julien N Jost

**Affiliations:** 1 Department of Neurosurgery, Kantonsspital Aarau, Aarau, CHE

**Keywords:** intracranial hypertension, hemicraniectomy, secondary decompressive craniectomy, brain contusion, primary decompressive craniectomy, cerebral hemorrhage, traumatic brain injury

## Abstract

Traumatic brain injuries (TBIs) still put a high burden on public health worldwide. Medical and surgical treatment strategies are continuously being studied, but the role and indications of primary decompressive craniectomy (DC) remain controversial. In medically refractory intracranial hypertension after severe traumatic brain injury, secondary decompressive craniectomy is a last resort treatment option to control intracranial pressure (ICP). Randomized controlled studies have been extensively performed on secondary decompressive craniectomy and its role in the management of severe traumatic brain injuries. Indications, prognostic factors, and long-term outcomes in primary decompressive craniectomy during the evacuation of an epidural, subdural, or intracerebral hematoma in the acute phase are still a matter of ongoing research and controversy to this day. Prospective trials have been designed, but the results are yet to be published. In isolated epidural hematoma without underlying brain injury, osteoplastic craniotomy is likely to be sufficient. In acute subdural hematoma (ASDH) with relevant brain swelling and preoperative CT signs such as effaced cisterns, overly proportional midline-shift compared to a relatively small acute subdural hematoma, and accompanying brain contusions as well as pupillary abnormalities, intraventricular hemorrhage, and coagulation disorder, primary decompressive craniectomy is more likely to be of benefit for patients with traumatic brain injury. The role of intracranial pressure monitoring after primary decompressive craniectomy is recommended, but prospective trials are pending. More refined guidelines and hopefully class I evidence will be established with the ongoing trials: randomized evaluation of surgery with craniectomy for patients undergoing evacuation of acute subdural hematoma (RESCUE-ASDH), prospective randomized evaluation of decompressive ipsilateral craniectomy for traumatic acute epidural hematoma (PREDICT-AEDH), and pragmatic explanatory continuum indicator summary (PRECIS).

## Introduction and background

Traumatic brain injury (TBI) is a public health challenge worldwide and a leading cause of mortality in young adults, as well as a major cause of death and disability across all ages in all countries [1]. After TBI, the development of a mass effect from traumatic parenchymal lesions, acute subdural hematoma (ASDH), or acute epidural hematoma (AEDH) can all lead to secondary brain injuries, permanent neurological deterioration, coma, or death [2].

Most patients after TBI with small parenchymal lesions, ASDH, or AEDH do not require urgent surgical evacuation. These patients are managed conservatively under strict neurological observation. Small hematoma and inconspicuous clinical status at admission do not exclude surgical treatment during the clinical course, since mass effect might evolve dynamically [3]. Progressive hematoma occurs in 50% of cases on computed tomography (CT) and is associated with elevation of intracranial pressure (ICP). Risk factors for progressive hematoma include male patients, older age, and coagulation disruption [4].

In cases of mass effect due to a hematoma and accompanying neurological deterioration, surgical evacuation with craniotomy or craniectomy is indicated. In craniotomy, replacement of the bone flap is performed. This approach does not require a second surgery, but the risk of subsequent ICP elevation is inherent. In craniectomy, the bone flap is left out after the evacuation of mass lesions to accommodate the expansion of the brain to control ICP. However, this procedure requires a second operation (cranioplasty). In primary decompressive craniectomy (DC), a large bone flap is directly excluded during the evacuation of a mass lesion in the acute setting. Secondary DC is a treatment modality in medically refractory posttraumatic ICP elevation in initially conservatively treated patients or in patients with primary osteoplastic craniotomy after the evacuation of a mass lesion and secondary refractory ICP elevation.

Secondary DC has been studied extensively in prospective randomized trials, but up to this day, indications for primary DC in TBI are not well defined for emergency surgery [5]. For example, there is no scoring system for the prediction of decision guiding [6].

A substantial part of patients in surgically treated TBI undergoes primary DC (up to 33%) [7]. Therefore, this literature review aims to provide an overview of clinically important criteria for deciding which patients are suitable candidates for primary DC after TBI and summarize data on long-term functional outcomes after primary DC.

## Review

Decompressive craniectomy

**Figure 1:**
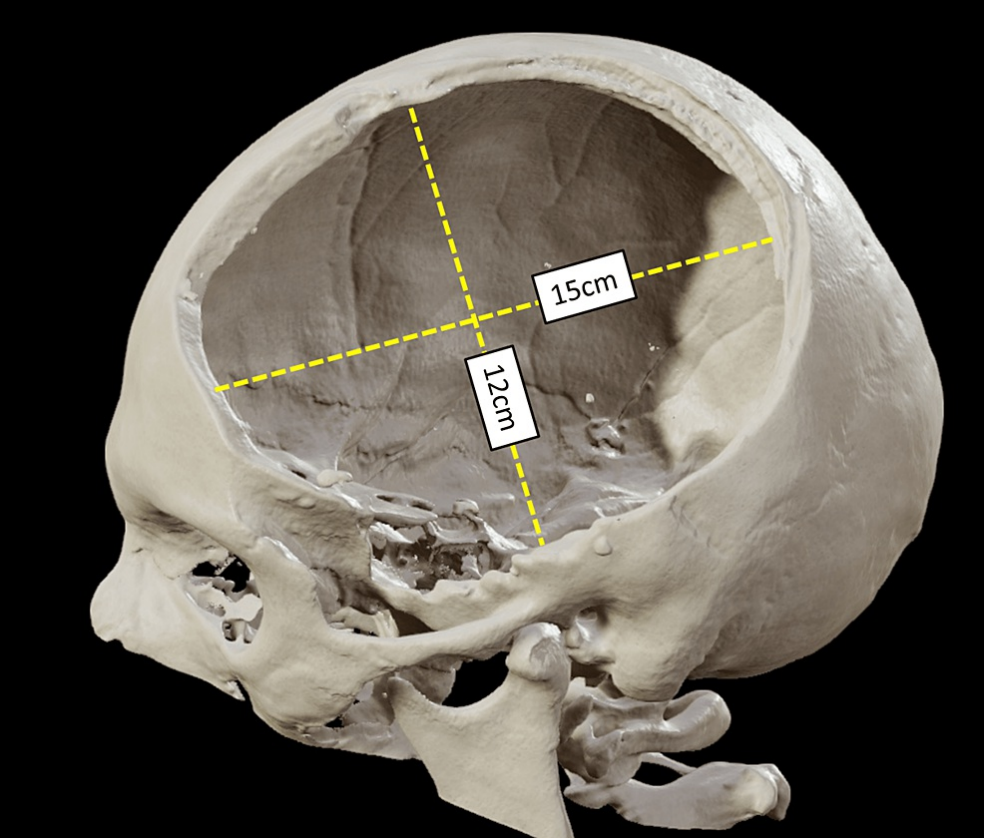
In DC, the bone flap needs to be at least 12 cm × 15 cm. It has been associated with better outcome. Authors' own creation/patient.

Several forms of DC are in use nowadays. These include bifrontal craniectomy and bilateral hemicraniectomy with diffuse brain swelling of both hemispheres. In unilateral swelling, unilateral frontotemporoparietal craniectomy or hemicraniectomy is performed. Any form of DC reduces ICP and increases brain tissue oxygen tension significantly. In DC, the bone flap should be large (at least 12 cm × 15 cm) and removal of bone to the middle cranial fossa floor is highly recommended since mortality and functional outcome were significantly better compared to a limited bone flap of 8 cm × 6 cm (Figure 1). Furthermore, delayed hematoma and cerebrospinal fistulas were significantly lower in the large bone flap group. The skin incision has to be larger than the bone flap and the dura must be opened followed by the implantation of an expansile dural graft. Optimal dural graft material, the role of contusion evacuation, the mode of bone flap storage, and the role of hinge craniotomy are still areas of uncertainty [8].

Primary versus secondary DC

Primary DC is leaving out the bone flap during the initial evacuation of intracranial mass lesions. Secondary DC is a treatment option for patients with diffuse, medically refractory posttraumatic cerebral edema and refractory intracranial hypertension in initially conservatively treated patients or in patients with primary osteoplastic craniotomy after the evacuation of a mass lesion and secondary refractory ICP elevation [9]. Research about secondary DC has been thoroughly performed in two prospective randomized trials named decompressive craniectomy (DECRA) and RESCUEicp [10]. RESCUEicp concluded at six months follow up, secondary DC in patients with TBI and refractory intracranial hypertension resulted in lower mortality but higher rates of vegetative state than conservative care [11].

Primary DC has not been studied as extensively as secondary DC, i.e., most of the research is retrospective. Nevertheless, it has been proposed that primary versus secondary DC are two different entities with their own patient characteristics, outcomes, and indications [12].

Primary DC in AEDH

The Brain Trauma Foundation (BTF) recommends craniotomy and evacuation of all AEDH with a volume of 30 ml or more independent of the Glasgow Coma Score (GCS). AEDH is most likely to have a low risk of intracranial hypertension following an evacuation due to the rare incidence of intraparenchymal injury [8]. Therefore, in isolated AEDH, primary DC is not recommended. In combined lesions with parenchymal lesions and brain swelling, research is not conclusive and an individual-based decision has to be taken. Primary DC after the evacuation of AEDH in deeply comatose patients was even associated with worse outcomes compared to craniotomy alone. However, selection bias might be a limitation in this retrospective study [13].

Primary DC in ASDH

The BTF recommends craniotomy and evacuation if ASDH is more than 10 mm thick or the midline shift is more than 5 mm independent of GCS. A decrease of GCS by two or more points to less than nine from the time of injury to hospital admission prompts a craniotomy, even if ASDH is less than 10 mm or midline shift is less than 8 mm. Asymmetric or fixed and dilated pupils are another indication for surgical treatment in ASDH [8].

We have no defined guidelines on whether to perform osteoplastic craniotomy or primary DC in these cases. Because ASDH is more associated with intraparenchymal lesions with a risk of brain swelling than AEDH [7], a high variation in the performance of primary DC in ASDH is noted worldwide. This variation is also believed to reflect the lack of high-quality evidence regarding the use of primary DC for ASDH evacuation [14]. The incidence of raised ICP above 20 mmHg in surgically treated ASDHs was reported to be up to 67% [15]. In another study, a sustained ICP peak greater than 45 mmHg was seen in 46% of surgically treated ASDH patients and was associated with significantly higher mortality [16]. ICP reduction seems to reduce mortality, but primary DC has been controversial in different studies.

Patients with ASDH and low GCS treated with osteoplastic craniotomy or primary DC showed no difference in the outcomes, but a higher mortality rate in the primary DC group in a retrospective series [17].

In a prospective review with 643 patients, of whom 243 received a primary DC, was associated with greater mortality and handicap rates [18]. Patients requiring primary DC have a higher risk of poor neurological outcomes compared to patients undergoing solely craniotomy or conservative treatment in TBI [19]. Another study found that primary DC in ASAH failed to show benefit in terms of neurological outcomes and should be reserved for cases with uncontrolled intra-operative brain swelling [20]. Primary DC was also associated with significantly higher in-hospital mortality after propensity score-matched analysis [21]. In a systematic review, primary DC was associated with worse clinical presentation and postoperative outcome compared with osteoplastic craniotomy [22]. Another study concluded that primary DC with the evacuation of intracranial hemorrhagic lesions was associated with worse functional outcomes in elderly patients with TBI [23]. A better functional long-term outcome in ASDH undergoing primary DC was detected whenever the initial GCS was more than four among adult patients [24].

Controversially, a prospective study suggests a more aggressive approach to the removal of bone flaps, even when ICP elevation is not a deciding factor in primary DC [25]. Similarly, a retrospective study showed that primary DC might be more effective than osteoplastic craniotomy in a selected patient population [26]. In a prospective, non-randomized trial, no significant differences were identified in short-term outcomes after limited craniotomy versus large primary DC for patients >65 years of age. The results indicate that primary DC can be accepted as a surgical treatment option for ASDH even in elderly patients [27]. DC is of benefit when performed <5 hours after injury in younger patients with Glasgow Coma Scale >5 [28].

Because of these controversial conclusions, the randomized evaluation of surgery with craniectomy for patients undergoing evacuation of acute subdural hematoma (RESCUE-ASDH) trial was designed. RESCUE-ASDH is the first randomized trial comparing primary DC versus craniotomy for adult head-injured patients with an ASDH. The results are yet to be published. Patient enrollment has been completed since 2019 with 463 patients in a period of 4.5 years [8].

In 2016, a study protocol for a prospective randomized evaluation of therapeutic decompressive craniectomy in severe traumatic brain injury with mass lesions (pragmatic explanatory continuum indicator summary - PRECIS) has been published. This study is assessing primary "prophylactic" DC against secondary "therapeutic" DC. The results are yet to be published [29].

Primary DC in intraparenchymal lesions

**Figure 2:**
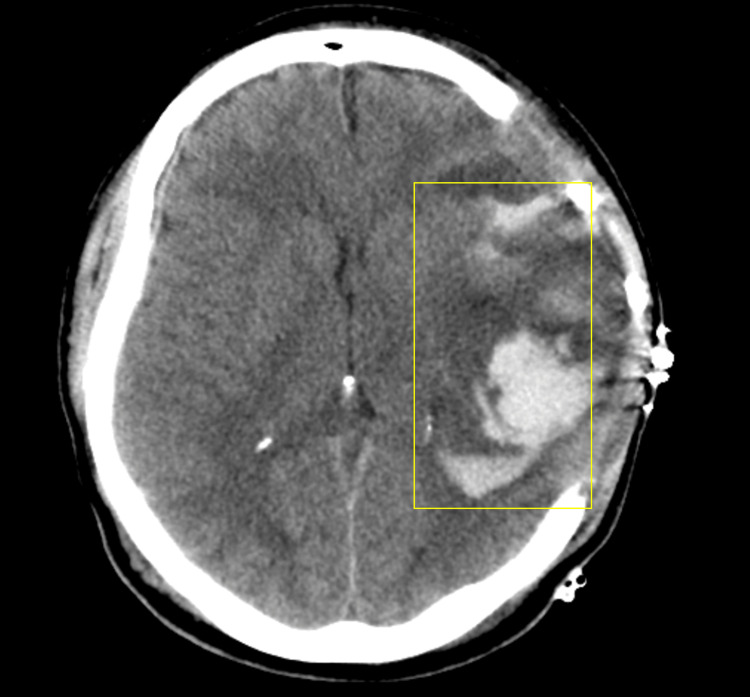
Postoperative intracerebral bleeding ("blooming or blossoming contusions") due to ICP reduction after DC is commonly seen and is associated with worse functional long-term outcome. Authors' own creation/patient.

Intraparenchymal lesions or contusions can occur in up to 35% of severe TBI. They significantly contribute to disability and death. Postoperative blooming contusions after DC are commonly seen as a complication in up to 75% (Figure 2). Predictors of contusion progression include initial GCS, history of hypertension, smoking, coagulopathy, contusion size, location, presence of subarachnoid hemorrhage (SAH), and ASDH [30]. Primary DC is recommended in mass effect due to brain swelling with brain contusions that are not being evacuated. Nowadays, it is still up to the surgeon's judgement whether to evacuate the intraparenchymal lesions [8]. Evacuation of progressing contusions in patients with deterioration in consciousness raises ICP and contusions larger than 30 mL is common practice [31]. Primary DC alone has been described as a safe and effective primary surgical intervention for the treatment of hemorrhagic contusion with the risk of further progression of intraparenchymal lesions [32]. Risk factors for the blossoming of hemorrhagic contusions include ASDH and a total volume of >2 ml before surgery. In patients who develop blossoming hemorrhagic contusion postoperatively after DC, the risk of an unfavorable outcome is increased [33].

Preoperative criteria for primary DC

**Figure 3:**
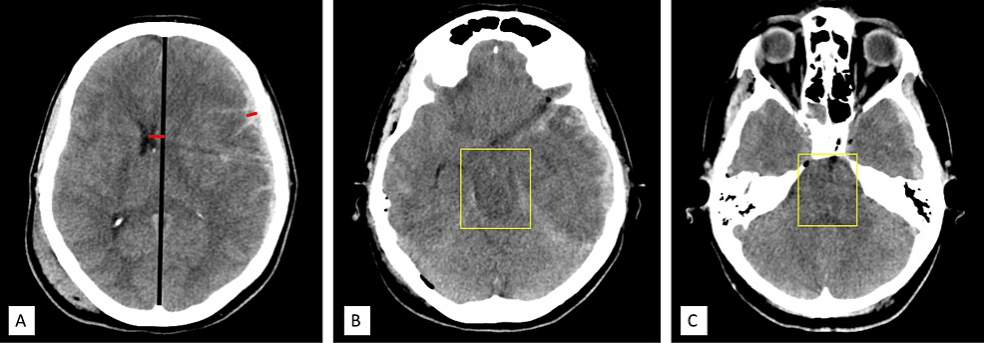
(A) Midline shift greater than hematoma thickness ratio; (B) and (C) effaced basal cisterns. Authors' own creation/patient.

A midline-shifting to maximal subdural hematoma thickness ratio of greater than one, intraventricular hemorrhage, and traumatic intracerebral hemorrhage on preoperative brain scans were identified as factors that primary DC is likely to be required [34]. Bilateral unresponsive pupil reaction and closed basal cistern were associated with primary DC selection in TBI with mass lesions, as these decreased the mortality and increased the functional outcome after six months. In one or two functioning pupillary reactions, mortality and functional outcomes rendered by GOS-E were not significantly different between the craniotomy group and the primary DC group. Patients without pupillary light reflex and primary DC compared to those with craniotomy had reduced mortality and more favorable functional outcomes [5] (Figure 3).

Intraoperative criteria for primary DC

Signs of intracranial hypertension intraoperatively, i.e., if the brain is bulging beyond the inner table of the skull intraoperatively, primary DC is recommended. If, however the brain is very relaxed after evacuation of ASDH or AEDH and preoperative CT did not show signs of parenchymal injury, the bone flap should be replaced [8]. Increased bleeding tendency intraoperatively was identified as factor that primary DC would be required [34].

Cranioplasty after primary DC

**Figure 4:**
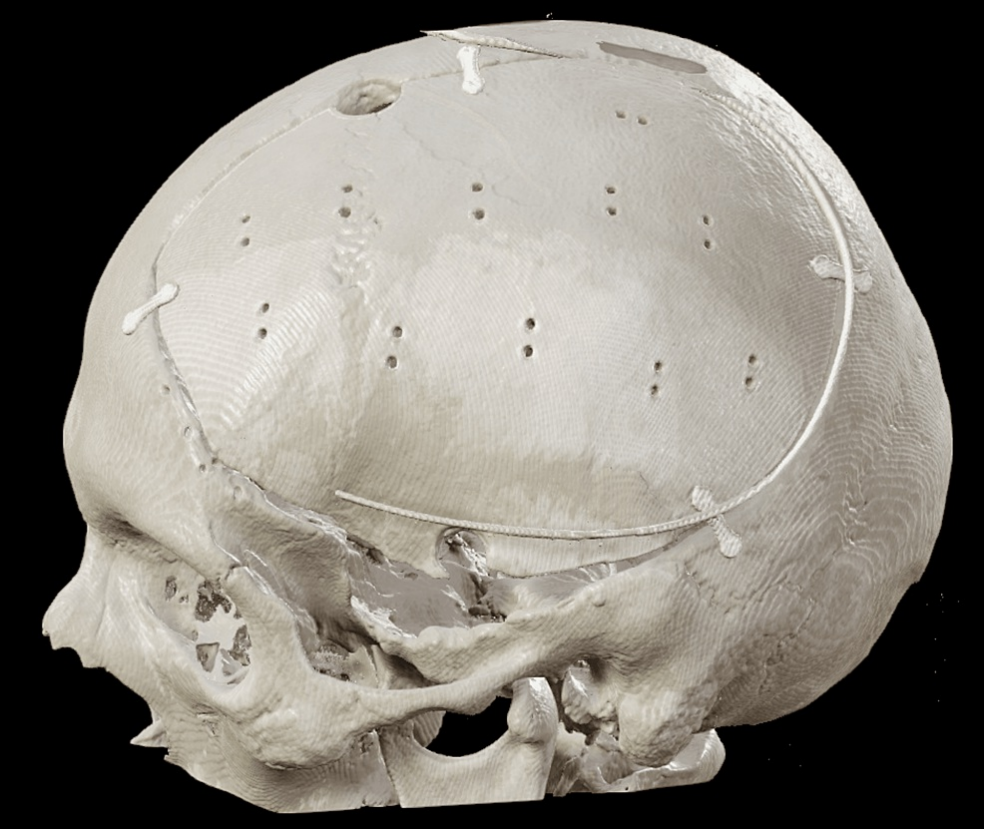
Cranioplasty. Authors' own creation/patient.

Cranioplasty or cranial reconstruction after primary DC is inevitable since brain protection and reconstruction of the cranial contour are necessary (Figure 4). Cranioplasty itself can improve neurological outcomes. It can restore cerebrospinal fluid dynamics and increases brain perfusion; this again can lower the risk of hydrocephalus and the incidence of the syndrome of the trephined. Last but not least, it increases the quality of life as it aids social interactions with a restored skull contour. Unfortunately, cranioplasty carries a high risk of postoperative complications such as bone resorption and surgical site infections. Overall complications have been reported between 10.9% and 40.4%. The optimal timing and material for cranioplasty are still uncertain. One systematic review showed a better neurological functional outcome in cranioplasty performed within three months.

Because of the above-mentioned complications, alternative methods, including hinge craniotomy or a four-quadrant osteoplastic decompressive craniotomy, were proposed. Both methods include the replacement of the bone flap with the possibility of expansion outward. In hinge craniotomy, the bone flap is introduced before closure, with one missing plate attachment on the bone edge, so outward expansion with prevention of sinkage inwardly of the bone flap is possible. Hinge craniotomy demonstrated ICP control compared to traditional DC [8].

In four-quadrant osteoplastic decompressive craniotomy, the bone flap is divided into four quadrants and the periosteum of the four pieces is sutured loosely to accommodate brain swelling [35]. In both techniques, class-I evidence is still missing.

ICP monitoring after primary DC

In osteoplastic craniotomy, an ICP monitor should be placed [8]. Regarding ICP monitoring after primary DC, several retrospective studies concluded its usefulness in guiding therapy postoperatively since intracranial hypertension and low cerebral perfusion pressure (CPP) occurred frequently after primary DC. Their occurrence is associated with an unfavorable neurological outcome [36]. Another study suggested the placement of an ICP monitor at the time of primary DC if intraoperative brain swelling is present [37]. Altogether, it was associated with significantly decreased in-hospital mortality [38]. If ICP monitoring is not available, serial CT scans are recommended for neurological monitoring [8]. In a retrospective study, the value of ICP monitoring in predicting re-operation using salvage DC was investigated. They found independent risk factors of DC, which are higher initial ICP, older age, early hypotension, and combined intracranial lesions. Primary DC might be a viable option in this patient group with objectively elevated intraoperative ICP [39]. Altogether, there is no class I evidence regarding ICP monitoring after primary DC.

Functional outcome and mortality after primary DC

In patients undergoing primary DC after traumatic brain injury, the predictors of 30-day mortality included older age, bilateral unreactive pupils, subdural hemorrhage, completely effaced basal cistern, intraoperative hypotension, coagulation disorder, and worse Injury Severity Score [6]. Overall mortality in primary DC was reported to be higher compared to a craniotomy group (15% vs. 5%) in patients undergoing surgery after TBI [17]. However, selection bias is probable since significantly more patients in the DC group had diffuse injury reported in preoperative CT scans and increased injury severity scores. One study found similar favorable functional outcome scores in the craniotomy and primary DC groups [6]. Controversially, another study concluded a higher risk for poor neurological outcomes in primary DC compared to craniotomy or conservative treatment alone. The poor prognosis is most likely related to the TBI severity itself rather than the intervention [19]. In more severe forms of TBI, complications such as hydrocephalus, subdural effusion, and outward herniation are more common and are associated with worse functional outcomes [40]. An acceptable functional long-term outcome after primary DC was found in 45.9% [41]. To aid clinical decisions, a risk prediction nomogram was developed to predict a six-month unfavorable outcome in patients undergoing primary DC after TBI. Similar independent predictors as mentioned before were identified. Higher age, lower GCS, effacement of cisterns, presence of coagulopathy, intraoperative hypotension, and intraoperative blood loss were all associated with worse functional long-term outcomes [42]. Decreased maximum cerebral perfusion pressure after primary DC was an independent indicator of worse outcomes and the probability of secondary DC [43]. Therefore, functional outcomes and mortality remain controversial in different studies. Further prospective studies are highly warranted.

## Conclusions

While secondary DC has been extensively studied with prospective, randomized trials, primary DC remains a challenge, especially in intermediate cases with ASDH and underlying brain injury. More refined guidelines and class I evidence will likely follow with the completion of the RESCUE-ASDH, prospective randomized evaluation of decompressive ipsilateral craniectomy for traumatic acute epidural hematoma (PREDICT-AEDH), and PRECIS trials. At the moment, the following recommendation has been described in the literature: Isolated epidural hematoma without underlying cerebral injury is best treated by craniotomy only. In ASAH with relevant brain swelling and preoperative CT-signs such as effaced cisterns, overly proportional midline-shift compared to a relatively small ASDH, and accompanying brain contusions as well as pupillary abnormalities, intraventricular hemorrhage, intraoperative signs of intracranial hypertension, and increased bleeding tendency, prompt for a primary DC rather than a craniotomy alone. ICP monitoring after primary DC is recommended in several retrospective studies. The role of hinge craniotomy to avoid a second surgery (cranioplasty) is yet to be studied. Prognostic factors and functional outcomes between primary DC and craniotomy alone remain controversial in different studies. In retrospective studies, primary DC was associated with higher morbidity and mortality. However, primary DC was more likely to be performed in patients with higher injury severity. Further high-quality, prospective studies are highly warranted.
